# A rapid culture system uninfluenced by an inoculum effect increases reliability and convenience for drug susceptibility testing of *Mycobacterium tuberculosis*

**DOI:** 10.1038/s41598-018-26419-z

**Published:** 2018-06-05

**Authors:** Yong-Gyun Jung, Hyejin Kim, Sangyeop Lee, Suyeoun Kim, EunJi Jo, Eun-Geun Kim, Jungil Choi, Hyun Jung Kim, Jungheon Yoo, Hye-Jeong Lee, Haeun Kim, Hyunju Jung, Sungweon Ryoo, Sunghoon Kwon

**Affiliations:** 1 Interdisciplinary Program of Biomodulation, Myongji University, Yongin, Gyeonggi-do 17058 Republic of Korea; 2 Myongji Bioefficiency Research Centre, Myongji University, Yongin, Gyeonggi-do 17058 Republic of Korea; 3 Center for Nutraceutical and Pharmaceutical Materials, Myongji University, Yongin, Gyeonggi-do 17058 Republic of Korea; 4 Korean Institute of Tuberculosis, Osong, Cheongju, Chung Buk 28158 Republic of Korea; 5 QuantaMatrix Inc., Seoul National University Hospital CMI, Jongno-gu, Seoul, 03082 Republic of Korea; 6 Department of Electrical Engineering and Computer Science, Seoul National University, Seoul, 08826 Republic of Korea; 7 Institutes of Entrepreneurial BioConvergence, Seoul National University, Seoul, 08826 Republic of Korea; 8 Seoul National University Hospital Biomedical Research Institute, Seoul National University Hospital, Seoul, 03080 Republic of Korea; 9 Clinical Research Centre, Masan National Tuberculosis Hospital, Changwon, Korea

## Abstract

The Disc Agarose Channel (DAC) system utilizes microfluidics and imaging technologies and is fully automated and capable of tracking single cell growth to produce *Mycobacterium tuberculosis* (MTB) drug susceptibility testing (DST) results within 3~7 days. In particular, this system can be easily used to perform DSTs without the fastidious preparation of the inoculum of MTB cells. Inoculum effect is one of the major problems that causes DST errors. The DAC system was not influenced by the inoculum effect and produced reliable DST results. In this system, the minimum inhibitory concentration (MIC) values of the first-line drugs were consistent regardless of inoculum sizes ranging from ~10^3^ to ~10^8^ CFU/mL. The consistent MIC results enabled us to determine the critical concentrations for 12 anti-tuberculosis drugs. Based on the determined critical concentrations, further DSTs were performed with 254 MTB clinical isolates without measuring an inoculum size. There were high agreement rates (96.3%) between the DAC system and the absolute concentration method using Löwenstein-Jensen medium. According to these results, the DAC system is the first DST system that is not affected by the inoculum effect. It can thus increase reliability and convenience for DST of MTB. We expect that this system will be a potential substitute for conventional DST systems.

## Introduction

Tuberculosis (TB) remains a major global health concern; it killed 1.5 million people in 2014^1^. The key to stopping TB transmission is rapid diagnosis and correct treatment with regimens based on drug susceptibility tests. To cope with and detect the emergence of resistant TB, much effort has been devoted to improving diagnostic tools based on culture and molecular techniques.

Molecular DSTs based on the detection of genotypic mutations are advantageous for the rapid screening of drug resistant TB, but there are critical gaps because the correlation of genotypic mutations with drug resistance are not fully understood and because the molecular DSTs have been applied only in some drug-resistant cases^2,3^. Additionally, these DSTs cannot detect all mutations involved in resistance, because the commercialized tests (line probe assays and Xpert MTB/RIF) only cover certain genes and regions (e.g. a limited resistance-determining region (RRDR) of the *rpoB* gene)^4^. Culture-based DSTs, called “phenotypic DSTs”, analyze viable cells grown either in broth or on solid medium and detect phenotypic resistance. The phenotype DST methods are widely accepted as a gold standard by the World Health Organization (WHO), but have not been widely performed owing to their disadvantages: (1) they are time-consuming, (2) they present a risk of potential infection and (3) the results of phenotype DST methods are not fully reproducible^2,5^.

MGIT 960 (Becton Dickinson, MD, USA) is a liquid-culture system that can shorten the DST running time from the 4~6 weeks of the Löwenstein-Jensen (L-J) method to ~13 days. Although MGIT 960 offers faster DST results than solid methods, it still has many barriers to overcome, as do another conventional methods^6,7^. First, one of the main barriers is preparing the proper cell number at the inoculum stage. The culture-based DST methods require a strict standardized inoculum size to produce reproducible DST results. However, in the MTB DST, it is not easy to evenly homogenize clumps of waxy-coated MTB cells; the clumps possibly cause inadequate cell numbers at the inoculum stage, leading to the inoculum effect (IE) during DST. IE is one of the major problems that cause DST errors, leading to reduced drug activity from the increased bacterial cell density^8–10^. Consequently, IE can increase the MICs of anti-TB drugs, resulting in irreproducible DST results. Secondly, the procedures involved in the inoculum preparation and culturing are always a concern from the standpoint of biosafety because the MTB cell suspension is serially diluted to adjust the correct cell density and this step could generate MTB aerosols, resulting in increased risk of laboratory TB transmission.

To date, there have been various approaches to shorten the turn-around time of DSTs, but none have attempted to overcome the inoculum effect even though it influences the reproducibility of DST results. Using microfluidics and imaging technologies, we designed and developed the Disc Agarose Channel system, which is fully automated and capable of tracking single cell growth to produce MTB DST results within 3~7 days^11^.

In the present study, we observed that this system is not influenced by inoculum size. To verify this finding, we evaluated the consistency of MIC values with various inoculum sizes in the range of ~10^3^ to ~10^8^ CFU/mL, as well as determined the critical concentration and validated the DAC system without measuring inoculum size on clinical isolates for clinical application. These results showed that the DAC system is not affected by inoculum size, and we observed strong agreement (96.3% overall agreement for the 12 tested anti-TB drugs) between the DAC system and the L-J method (reference method).

## Materials and Methods

### Strains

The *M*. *tuberculosis* H37Rv ATCC 27294 and total 484 clinical MTB strains containing Pan-susceptible, multidrug resistant (MDR) and extensively drug resistant (XDR) isolates were obtained from the Korean Institute of Tuberculosis (KIT). All procedures for MTB cultures and DSTs were performed using a biological safety cabinet (BSC) inside the KIT’s biosafety level 3 (BSL3) laboratory. Drug susceptibility results of all clinical isolates in this study were previously determined by the absolute concentration method using L-J medium prepared in-house^12,13^.

### Analysis of activities of anti-TB drugs with various inoculum concentrations

For the inoculum effect study, all isolates were freshly sub-cultured on L-J medium before being used. The colonies were vortexed in a tube containing a few drops of PBS and glass beads, to break up the large clumps. The bacterial cells were inoculated as a set 5 × 10^3^~5 × 10^8^ CFU/mL including the standard inoculum of bacterial concentration (~5 × 10^5^ CFU/mL). To create high bacterial density (~10^8^ CFU/mL), the dense suspension was centrifuged for 10 minutes at 3,000 *g* and the pellet was resuspended in sterile PBS to achieve ~10^8^ CFU/mL. Then, this bacterial suspension was serially diluted. The cell numbers were counted by the bacterial CFU method on Middlebrook 7H11 agar (BD BBL, MD, USA) plates with the easySpiral plater system (Interscience, Saint Nom la Breteche, France). To estimate the MIC values for the 4 first-line drugs, five concentrations were tested according to two-fold serial dilutions. MTB H37Rv and two clinical isolates were prepared as described above. The MICs for each drug were estimated with various inoculum sizes, ranging from ~10^3^ to ~10^8^ CFU/mL. To compare the appropriate inoculum size among three DST tests, L-J DST and MGIT 960 DST were both performed by the standard methods according to a previously described study^14,15^. The DSTs of three different methods with H37Rv were performed with differing inoculum sizes, ranging from ~10^4^ to ~10^7^ CFU/mL^16^. The critical concentrations (CCs) of the DAC system were adopted as the breakpoints of the BACTEC 460 TB and MGIT 960 systems, based on the Middlebrook 7H9 (BD BBL, MD, USA) broth, because it was reported that the CCs were dependent on the medium^17^, and the DAC system was also based on the Middlebrook 7H9 broth. The CCs of the DAC system were 0.1 μg/mL for INH, 1.0 μg/mL for RIF, 1.0 μg/mL for STR, and 5.0 μg/mL for EMB.

### Broth microdilution test

The broth microdilution (BMD) test was used as a gold standard recommended by the Clinical and Laboratory Standards Institute (CLSI)^18^. The extended spectrum beta-lactamase (ESBL)-negative *E*. *coli* ATCC 25922 strain was purchased from ATCC and clinical the ESBL-positive *E*. *coli* was obtained at SNUH as described previously^19^. For the BMD test, the cefepime solutions were prepared from the stock solution. A 100 μL volume of the antimicrobial agent at the appropriate concentration, which was determined by the CLSI recommendation^18^, was pipetted into the bottom of 96-microwell plates (Falcon/BD Biosciences, CA, USA). Ten microliters of bacterial stock solution was inoculated into the wells at final concentrations of 10^5^~10^7^ CFU/mL. After 16~20 h of incubation at 37 °C, the MIC values of the microdilution wells were read as the concentration at which there was ≥99% reduction in growth compared to the control, by unaided visual inspection.

### Drug susceptibility test

**Figure 1:**
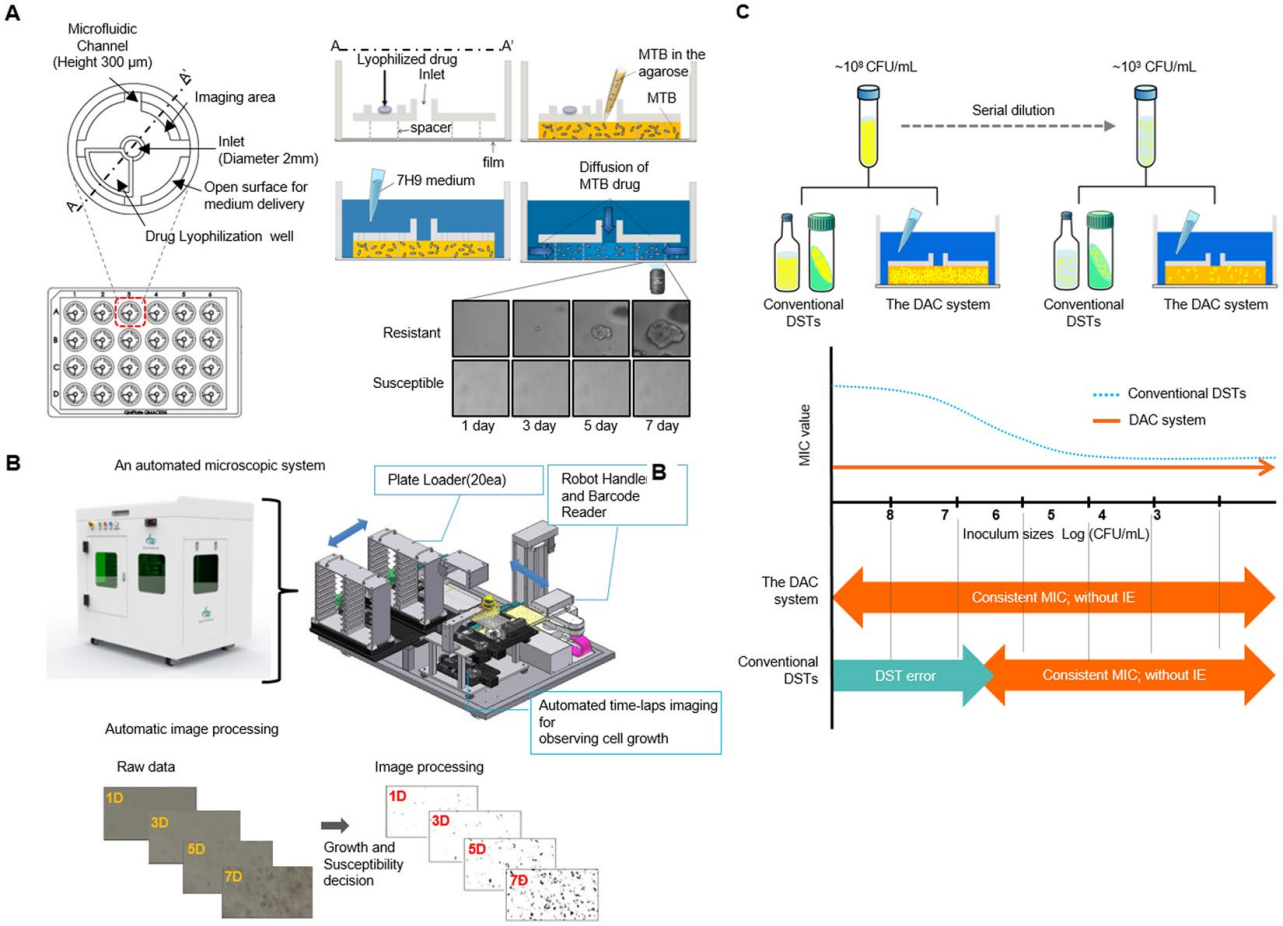
The fully automated DAC system performs DST-based MTB imaging and has no inoculum effect, leading to reliable DST results. **(A)** Schematic of the DAC chip containing 12 anti-TB lyophilized drugs. The detailed methods for DAC DST are as described in the Materials and Methods. **(B)** In an automated microscopic system, the DAC chip was loaded and unloaded on the reader and one area at the edge of the agarose was automatically imaged every other day using the time-lapse method. Then, growth images were automatically processed. **(C)** Advantages of the DAC system compared with conventional solid and liquid DST systems for MTB: there are inoculum effects over ~10^6^ CFU/mL in the conventional DST systems that can cause DST errors, but no inoculum effect is observed in the DAC system, leading to consistent MIC values regardless of inoculum sizes from ~10^3^ CFU/mL to ~10^8 ^ CFU/mL.

The DST method of the DAC system was previously described^11^. We mixed 300 μL of the MTB cell suspension with 900 μL of 0.5% agarose at 37 °C by vortexing. Subsequently, 40 μL of 0.375% agarose mixture with MTB cell suspension was loaded into the inlet of a DAC chip. The agarose was then allowed to solidify at room temperature for 1 minute. Each TB drug was lyophilized and added into each well. The lyophilized drug resolved immediately after a 0.5 mL addition of the 7H9 broth containing 10% OADC; the proper concentration of each drug was adjusted. The resolved drug in the culture medium was then allowed to diffuse into the agarose. After this process, the DAC chip was then sealed by an air-permeable film for safety and prevention of contamination and incubated in a temperature-controlled culture chamber at 37 °C for 7 days. One area at the edge of the agarose was automatically imaged with a 20 × lens on an inverted microscope every other day using the time-lapse method. Growth images were then automatically processed (Fig. 1).

### Determination of critical concentrations in the DAC system

Based on the critical concentrations of the BACTEC 460 TB and MGIT 960 systems for each drug recommended by the WHO policy guidelines on DST of second-line anti-TB drugs in 2008, five concentrations were chosen according to two-fold serial dilutions. Strains were chosen from 89 well-characterized susceptible groups obtained from new cases and 141 resistant groups obtained during drug treatment from pulmonary tuberculosis patients (Supplementary Table S1). After graphing the curve of MIC distribution, the CCs for each drug were determined at the concentration where the cumulative percentage difference between susceptible and resistant strains mostly showed the greatest percentage difference as previously described^13^.

### Validation of the DAC system

The DAC system established CCs was validated with a total of 254 clinical MTB strains including pan-susceptible strains and resistant strains tested by the L-J method. Each drug was lyophilized in the DAC chip according to its critical concentration. To ensure the consistency of lyophilized drugs, the reference strain MTB H37Rv ATCC 27294 and the clinical isolate KIT87190 strain were used as internal controls for each test of DST^20^. DSTs of the DAC system were performed without measuring the MTB inoculum size and the DST results of the DAC system were compared with those of the L-J method. For a blinded assessment of the outcomes, results from the DAC system were determined automatically using an imaging processing program without knowledge of the results from the reference method.

### Resolution of discrepancy between the DAC system and the L-J method

The discrepant results between the DAC system and the L-J method were confirmed by DNA sequence analysis^21,22^. We performed DNA sequencing by using the target regions involved in the following genes as previous described^22^; *rpoB* (RRDR) for rifampin and rifabutin, *embB* for ethambutol, *rrs* and *rpsL* for streptomycin, *gyrA* for quinolones, and *rrs*, *eis* and *tlyA* for kanamycin, amikacin and capreomycin, respectively. Target regions for each gene are *rpoB* = 759807–763325, *embB* = 4246517–4249810, *rrs* = 1471846–1473382, *rpsL* = 781560–781934, *gyrA* = 7302–9818, *eis* = 2714124–2715477, and *tlyA* = 1917940–1918746 of the H37Rv genome sequence (GenBank accession number NC_000962.3)^22^.

### Antimicrobial agents and drug lyophilization in the DAC chip

All drugs were purchased from Sigma-Aldrich (St. Louis, MO, USA) including the first line drugs (isoniazid (INH), rifampin (RIF), streptomycin (STR), ethambutol (EMB), and rifabutin (RFB)) and the second-line drugs (amikacin (AMI), capreomycin (CAP), kanamycin (KAN), levofloxacin (LEV), moxifloxacin (MOXI), ofloxacin (OFL), and para-aminosalicylic acid (PAS)). Stock solutions of each antibiotic were prepared in accordance with the manufacturer’s instructions and working solutions were prepared fresh from the stock solution. For drug lyophilization, all drugs in the DAC chip were freeze-dried under a vacuum, and the freezing process was carried out using a freeze dryer (Ilshin Biobase Co., LTD., Korea). After freeze-drying, the DAC chip was packaged in an aluminum foil vacuum-sealed pouch and stored at 4 °C before use. The 12 lyophilized anti-TB drugs in the DAC chip were validated by MIC determination using the clinical isolate KIT87190 strain. Quality control ranges of MIC value for each drug were determined from three different batches twice a week for 5 weeks, resulting in 30 replicates in total (Supplementary Table S2)^23^.

## Results

### Activities of anti-TB drugs in the DAC system are not affected by inoculum size

**Figure 2:**
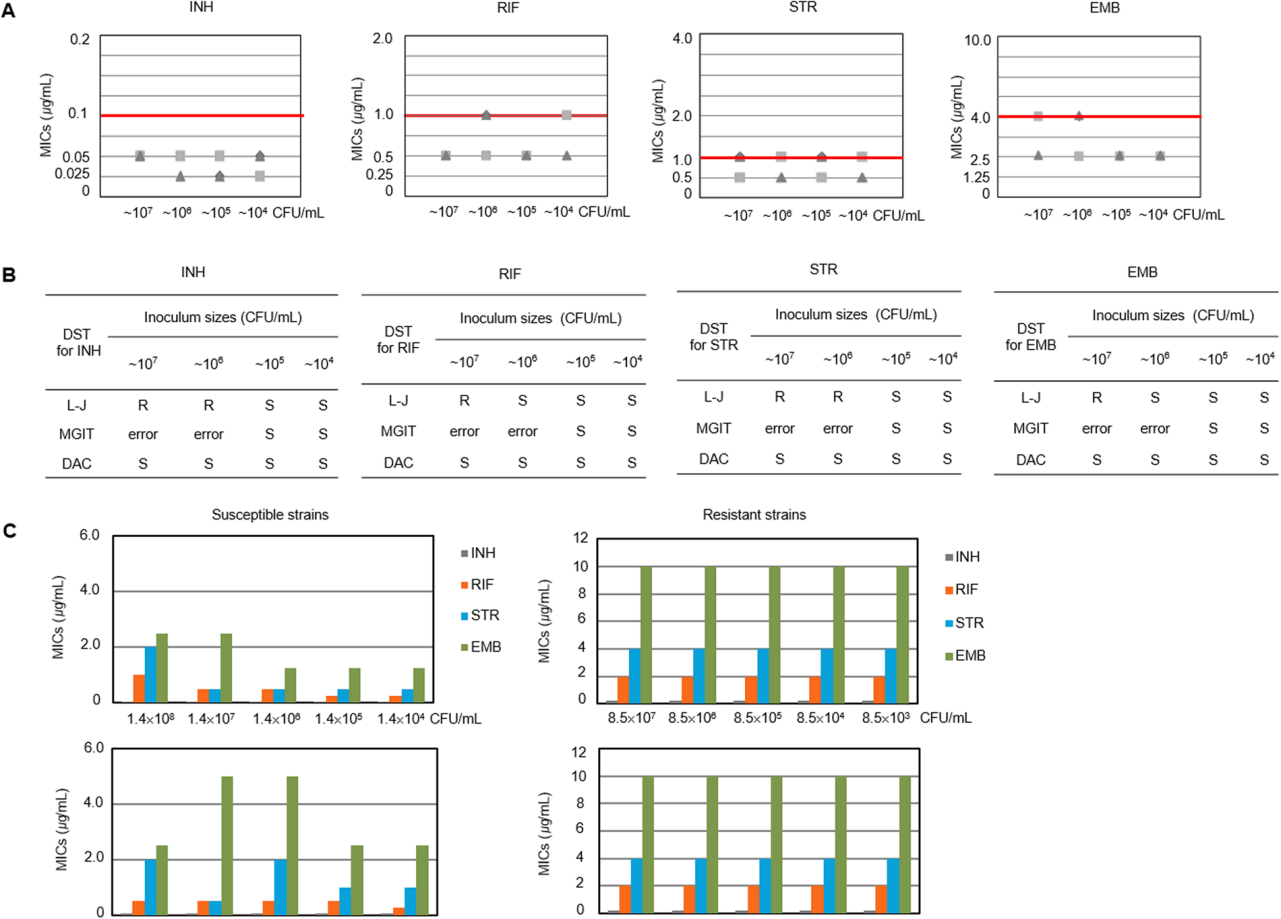
Activities of anti-TB TB drugs in the DAC system are not affected by inoculum size. **(A)** The MIC values for the first-line drugs according to various inoculum sizes. MTB H37Rv ATCC 27294 cells from ~10^4^ to ~10^7 ^CFU/mL were inoculated in the DAC system and the MIC values were determined. The spots (circle, triangle and square) of each drug indicate the MICs values from three independently repeated experiments. The tested concentrations for each drug were a two-fold dilution scale. The breakpoints of the BACTEC 460 TB and MGIT 960 systems based on the Middlebrook 7H9 broth were adopted for the DAC system; 0.1 μg/mL for INH, 1.0 μg/mL for RIF, 1.0 μg/mL for STR, and 5.0 μg/mL for EMB. The red horizontal line indicates the breakpoints for each drug. All MIC values were determined under the breakpoints. **(B)** The comparison of an inoculum effect for the first-line drugs against H37Rv between the DAC system and two routine methods, the L-J method (solid) and MGIT 960 method (liquid). The various inoculum sizes from ~10^4^ to ~10^7^ CFU/mL were tested. The DST results were represented as resistant (R) or susceptible (S). The DST results were consistently “S” regardless of the various inoculum sizes in the DAC system, whereas they were changed from “S” to “R” or “Error” over 5 × 10^6^ CFU/mL in the two routine methods. **(C)** The MICs values from clinical isolates in the various inoculum sizes. The MIC values from two pan-susceptible and two resistant strains were estimated for the first-line drugs. The inoculum sizes were from ~10^3^ CFU/mL to ~10^8^ CFU/mL for two drug susceptible strains and two drug resistant strains. There was no inoculum effect with the clinical isolates in the DAC system.

Because the DAC system is a new method based on microfluidics and imaging technologies, the optimal bacterial density at the inoculum step needed to be determined by comparison with the MIC values from the conventional DST methods. First, we estimated the MICs for the first-line drugs (INH, RIF, STR and EMB) with the various inoculum sizes in the range of ~10^4^ to ~10^7^ CFU/mL. The MIC values from the different inoculum sizes of MTB H37Rv, the standard laboratory strain, were determined and compared within 7 days. Surprisingly, consistent MICs were observed with essential agreement regardless of the inoculum size: The MIC values were 0.025 μg/mL and 0.05 μg/mL for INH, 0.5 μg/mL and 1.0 μg/mL for RIF, 0.5 μg/mL and 1.0 μg/mL for STR, and 2.5 μg/mL and 5.0 μg/mL for EMB with inoculum sizes of ~10^4^ to ~10^7^ CFU/mL, respectively (Fig. 2A).

To compare the DAC systems with the other conventional DST systems on the point of the inoculum effect, the DSTs of three different methods with H37Rv were performed with various inoculum sizes from ~10^4^ to ~10^7^ CFU/mL. The DST results of the DAC system with H37Rv were all “susceptible (S)” regardless of the inoculum size. In contrast, the DST results of either the L-J method or MGIT 960 were “resistant (R)” or “system error” when the inoculum size was higher than ~10^6^ CFU/mL (Fig. 2B). This result indicated that the DAC system was not sensitive to the inoculum effect, in contrast to the other conventional DST methods.

For further verification, we examined four clinical isolates, two pan-susceptible strains and two XDR MTB strains. In the DAC system, we did not observe any inoculum effect from MIC determinations of the clinical strains with various inoculum sizes from ~10^3^ to ~10^8^ CFU/mL (Fig. 2C). The MIC values were determined with essential agreement regardless of inoculum size, with the exception of STR against two susceptible strains. The MIC values of STR were from 0.5 to 2.0 μg/mL, but the highest value did not exceed 2.0 μg/mL, which was the critical concentration of the BACTEC 460 TB system^24,25^.

**Figure 3:**
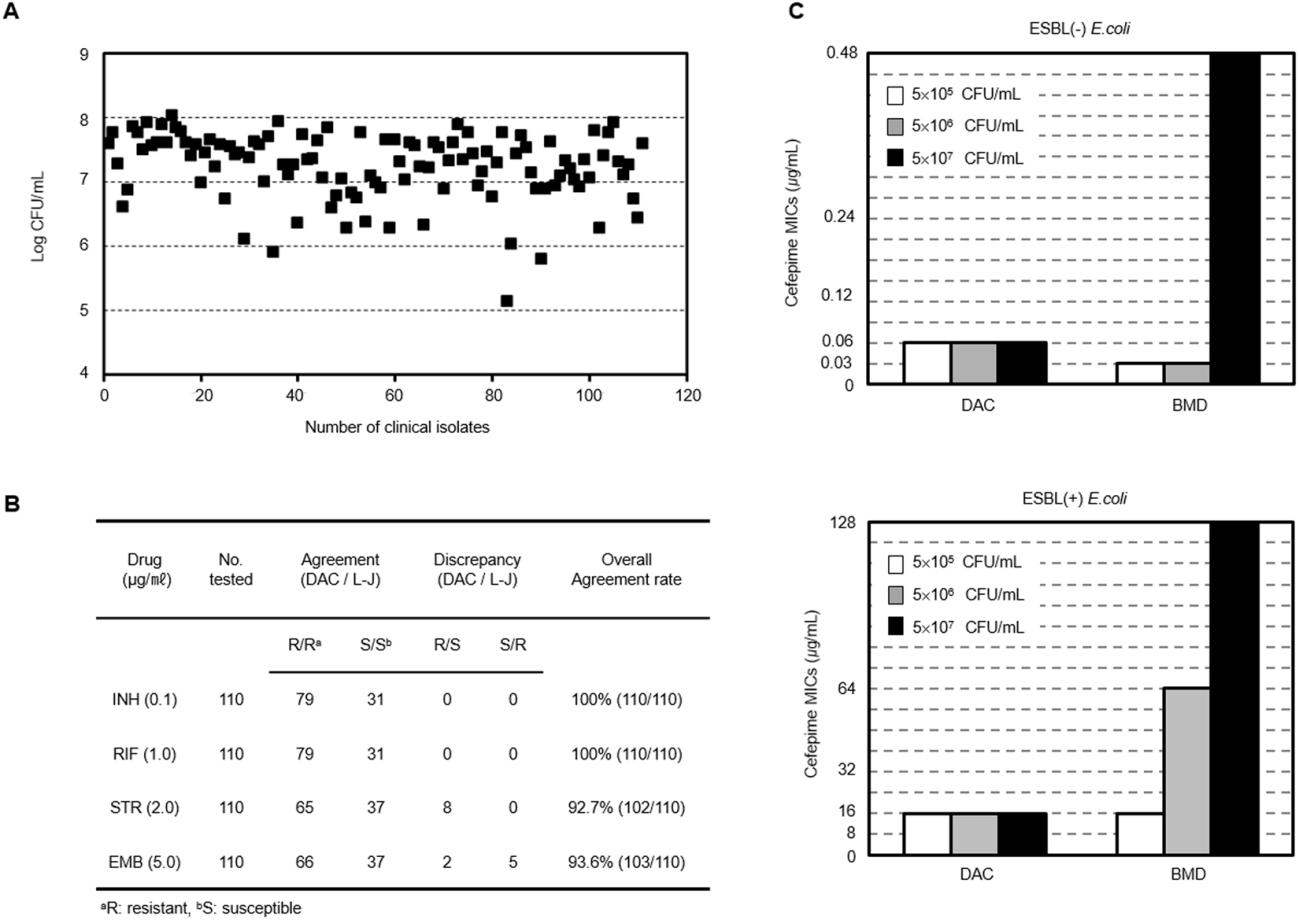
DSTs with the randomly prepared inocula of clinical isolates. **(A)** The distribution of random inoculum sizes prepared without measuring cell density. Four researchers randomly prepared 110 clinical isolates, and the bacterial CFUs were counted afterward. The highest inoculum size was 1.1 × 10^8^ CFU/mL and the lowest was 1.4 × 10^5^ CFU/mL. **(B)** DST results with the randomly prepared inocula with clinical isolates. The DSTs for the first-line drugs were performed with the randomly prepared inocula in the DAC system, and the DST results were compared with those of the L-J method. There were high agreement rates between the two systems. **(C)** More severe inoculum effect in the ESBL-positive strain. Antibiotic-inactivating enzymes in the culture medium caused a more severe inoculum effect with respect to the use of cefepime according to the inoculum size. In the broth microdilution method, the inoculum effect in the ESBL-negative *E*. *coli* ATCC 25922 strain was over 10^7^ CFU/mL, and the inoculum effect of the ESBL-positive *E*. *coli* strain was over 10^6^ CFU/mL. However, in the DAC system, there was no inoculum effect with either the ESBL-negative or ESBL-positive strains from 10^5^ CFU/mL to 10^7^ CFU/mL.

For verification with extended clinical strains and random inoculum sizes, 110 clinical strains containing 31 pan-susceptible and 79 MDR MTB determined by the L-J method were tested. The DST results of the first-line drugs were determined and compared to those of the L-J method. The breakpoints of the BACTEC systems were employed as in Fig. 2B. The inoculums of all strains were randomly prepared without measuring cell density by four researchers. Then, 3 weeks afterward, they were counted by the bacterial CFU method on 7H11 agar plates. The highest inoculum size was 1.1 × 10^8^ CFU/mL, and the lowest was 1.4 × 10^5^ CFU/mL (Fig. 3A) according to the CFU counting results. In spite of various inoculum sizes with ~1,000-fold ranges, there were high agreement rates (overall 95.2% agreement) compared with the DST results of the L-J method (Fig. 3B).

With such consistent MIC data, the DAC system could minimize the inoculum effect that causes limitations in the *in vitro* drug susceptibility test^26,27^. To explain this phenomenon, the physical characteristics of the DAC system were considered. The enclosed environmental conditions of the agarose matrix may contribute to the minimization of the inoculum effect. To verify this hypothesis, the ESBL-negative *E*. *coli* ATCC 25922 and a clinical ESBL-positive *E*. *coli* strain were tested with inoculum concentrations of 5 × 10^5^ to 5 × 10^7^ CFU/mL and 0.015~128 μg/mL of cefepime (a beta-lactam antibiotic) in both the DAC system and the conventional BMD method. Interestingly, antimicrobial susceptibility tests against both ESBL-positive and -negative strains showed an inoculum effect for the BMD method, but not for the DAC system. The IE was more serious in the case of the ESBL-positive strain in the BMD method (Fig. 3C). This effect implies that in the BMD method, metabolites or proteins (beta-lactamase) produced by the ESBL-positive strain easily bind and inactivate cefepime, whereas in the DAC system, they could be trapped in the agarose, and cannot bind and inactivate the antibiotic, even at ~5 × 10^7^ CFU/mL.

### Determination of the critical concentration of anti-TB drugs in the DAC system

The CCs of five of the first-line and seven of the second-line anti-TB drugs in the DAC system were determined with 230 clinical isolates whose DST results were already well characterized by both the absolute L-J method and DNA sequence analysis. The inocula of all clinical strains were randomly prepared without measuring cell density.

**Figure 4:**
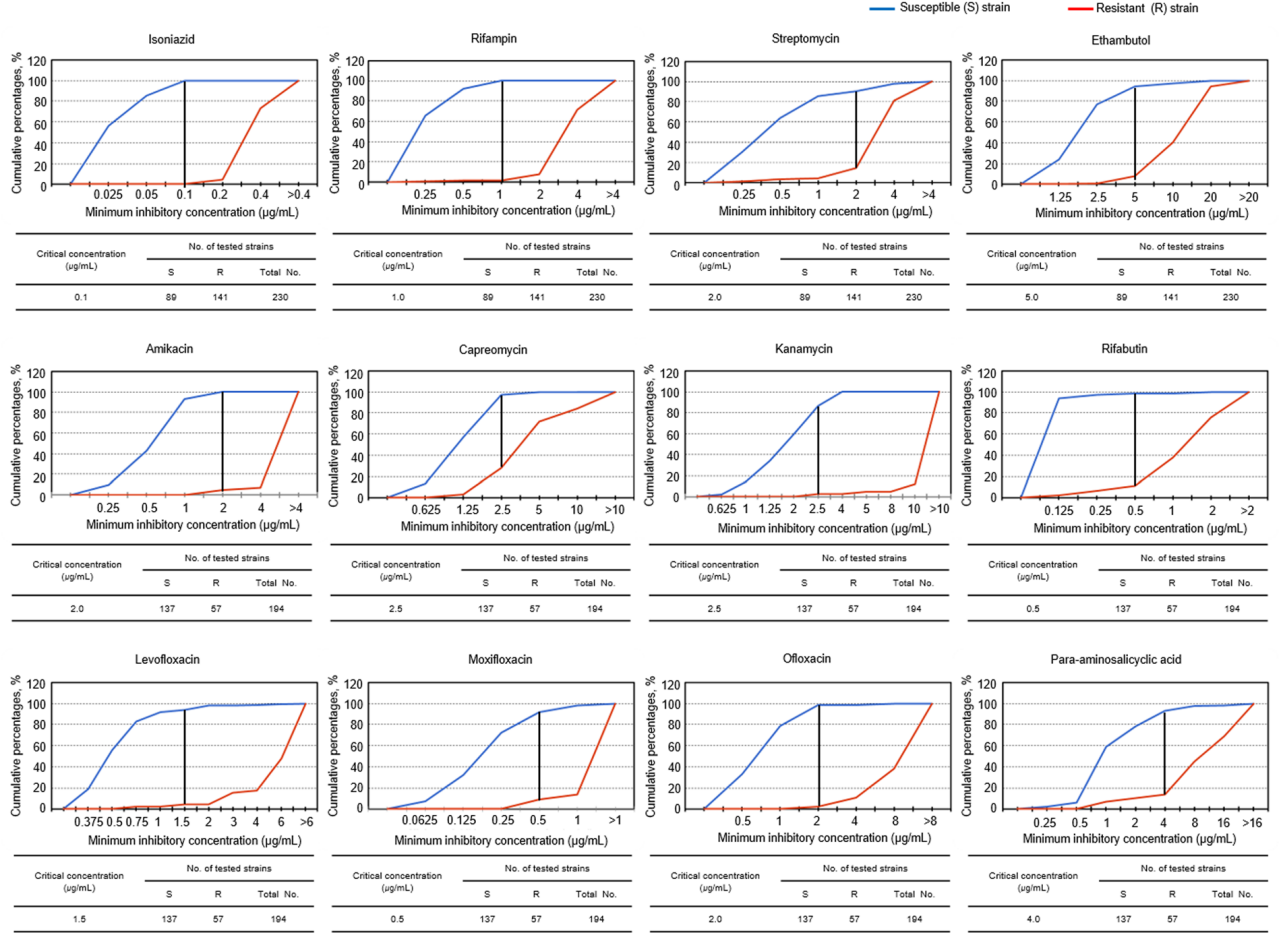
Determination of critical concentrations in the DAC system. Cumulative percentage plots of susceptible and resistant strains against respective MIC (μg/mL) values for each drug in the DAC system were obtained. Critical concentrations for 12 anti-TB drugs were determined mainly on the concentrations showing the greatest percentage difference. The numbers of the tested susceptible and resistant strains for each drug were shown in the table. The black number on the right side of the vertical line on the critical concentration shows the greatest percentage difference between susceptible strains and resistant strains, except STR and RFB.

The MIC values for the first-line drugs (INH, RIF, STR, EMB, and RFB) and the second-line drugs (AMI, CAP, KAN, LEV, MOXI, OFL, and PAS) were determined within 7 days. After determining the MIC of each drug, the CCs for each drug were established at the concentration where the cumulative percentage difference between susceptible and resistant strains mostly showed the greatest percentage difference except for STR and RFB (Fig. 4). The determined CCs were 0.1 μg/mL for INH, 1.0 μg/mL for RIF, 2.0 μg/mL for STR, 5.0 μg/mL for EMB, 2.0 μg/mL for AMI, 2.5 μg/mL for CAP, 2.5 μg/mL for KAN, 1.5 μg/mL for LEV, 0.5 μg/mL for MOXI, 2.0 μg/mL for OFL, 0.5 μg/mL for RFB, and 4.0 μg/mL for PAS.

For the CC of STR, there was the greatest percentage difference at 1.0 μg/mL. However, it was reported that the MIC range of STR against the MTB H37Rv and H37Ra by the BACTEC system were 0.094~0.75 and 0.38~1.5 μg/mL, respectively^28^, and the CC of STR was 2.0 μg/mL in the BACTEC system^24,25^. In addition, the MIC values of STR against the susceptible MTB strains isolated from new patients before drug treatment showed 1.0 or 2.0 μg/mL repeatedly in the DAC system. For these reasons, we determined that the CC of STR was 2.0 μg/mL in the DAC system. In the case of RFB, 0.125 μg/mL showed the greatest percentage difference, but 0.5 μg/mL was determined as the critical concentration according to the BACTEC system because the CC determination is dependent on the medium and both the DAC system the BACTEC system used the same 7H9 broth. There was no significant difference in the agreement rate between 0.125 μg/mL (96.9%) and 0.5 μg/mL (94.8%).

### Validation of the automated DAC system

In the previous work, the DAC system was fully automated with an image-processing program for rapid DST. The DSTs of the first-line drugs were performed with H37Rv and ~30 clinical isolates^11^. In this work, to create a more convenient system, each drug was lyophilized in the DAC chip, and the activities of lyophilized drugs showed consistency within quality control ranges of 12 anti-TB drugs at 4 °C for 6 months (Table 1 and Supplementary Table S2). Additionally, we obtained reproducible results for each drug, between tests done during different weeks by three different operators (Supplementary Table S3). To validate the CCs of this system in a clinical setting, DSTs of the first-line and second-line drugs were performed with 254 clinical isolates including susceptible and resistant MTB strains categorized by the L-J method. The inocula of all clinical strains were randomly prepared without measuring cell density. The DST results were compared with those of the reference method (the L-J method). INH and RIF showed very high agreements (100% and 99.6%, respectively) for both susceptible and resistant strains. The agreement rates for the other anti-TB drugs were also high, in the range of 91.3 ~ 99.2%. The overall agreement rate for all drugs was 96.3% (Table 2). Sixty-seven strains showed discrepant results between the DAC and the reference method. To resolve any discrepancy between-tests, we analyzed some discrepant results using DNA sequence analysis^21,22^. The comparison of results after their resolution is shown in Table 3. The DNA sequencing results of the target genes showed a better correlation when using the DAC system (62.1%) than when using the L-J method (37.9%). Although the L-J method has been considered the gold standard for phenotypic DST for MTB, they may show falsely low MICs, causing the resistant strain to be evaluated as susceptible to the tested drug, compared with the molecular DST results of whole-genome sequencing^29^, suggesting that the DAC system provides more accurate DST results than the conventional method.

**Table 1:** Validation of the 12 lyophilized anti-TB drugs.

Month	Drug	INH	RIF	STR	EMB	AMI	CAP	KAN	LEV	MOXI	OFL	PAS	RFB
	CC (μg/mL)	0.1	1.0	2.0	5.0	2.0	2.5	2.5	1.5	0.5	2.0	4.0	0.5
	Quality control range	0.025~0.05	0.125~0.5	0.25~1.0	1.25~2.5	0.25~1.0	0.625~2.5	0.625~2.5	0.375	0.0625~0.25	0.5~1.0	1.0~4.0	0.125~0.25
1	Fresh (1~3)	0.05	0.25	0.5	5.0	1.0	0.625	1.25	0.375	0.125	1.0	1.0	0.125
		(S)	(S)	(S)	(S)	(S)	(S)	(S)	(S)	(S)	(S)	(S)	(S)
	1	0.05	0.25	0.5	2.5	1.0	2.5	2.5	0.375	0.125	1.0	1.0	0.125
		(S)	(S)	(S)	(S)	(S)	(S)	(S)	(S)	(S)	(S)	(S)	(S)
	2	0.025	0.25	0.25	2.5	1.0	1.25	2.5	0.75	0.125	1.0	1.0	0.125
		(S)	(S)	(S)	(S)	(S)	(S)	(S)	(S)	(S)	(S)	(S)	(S)
	3	0.025	0.25	0.5	5.0	1.0	2.5	2.5	0.375	0.25	1.0	1.0	0.125
		(S)	(S)	(S)	(S)	(S)	(S)	(S)	(S)	(S)	(S)	(S)	(S)
2	Fresh (1~3)	0.05	0.25	0.25	2.5	0.25	0.625	1.25	0.375	0.125	0.5	1.0	0.125
		(S)	(S)	(S)	(S)	(S)	(S)	(S)	(S)	(S)	(S)	(S)	(S)
	1	0.05	0.25	0.25	2.5	0.5	1.25	1.25	0.375	0.125	0.5	1.0	0.125
		(S)	(S)	(S)	(S)	(S)	(S)	(S)	(S)	(S)	(S)	(S)	(S)
	2	0.025	0.25	0.25	2.5	0.5	0.625	1.25	0.375	0.125	0.5	1.0	0.125
		(S)	(S)	(S)	(S)	(S)	(S)	(S)	(S)	(S)	(S)	(S)	(S)
	3	0.05	0.25	0.25	2.5	0.5	0.625	1.25	0.375	0.125	0.5	1.0	0.125
		(S)	(S)	(S)	(S)	(S)	(S)	(S)	(S)	(S)	(S)	(S)	(S)
3	Fresh (1)	0.025	0.5	1.0	2.5	0.5	1.25	2.5	0.375	0.125	0.5	1.0	0.125
		(S)	(S)	(S)	(S)	(S)	(S)	(S)	(S)	(S)	(S)	(S)	(S)
	Fresh (2,3)	0.025	0.25	0.25	2.5	0.5	1.25	2.5	0.375	0.125	0.5	1.0	0.125
		(S)	(S)	(S)	(S)	(S)	(S)	(S)	(S)	(S)	(S)	(S)	(S)
	1	0.05	0.5	1.0	5.0	0.5	1.25	1.25	0.375	0.0625	0.5	1.0	0.125
		(S)	(S)	(S)	(S)	(S)	(S)	(S)	(S)	(S)	(S)	(S)	(S)
	2	0.025	0.25	0.25	2.5	0.25	0.625	1.25	0.375	0.0625	0.5	1.0	0.125
		(S)	(S)	(S)	(S)	(S)	(S)	(S)	(S)	(S)	(S)	(S)	(S)
	3	0.025	0.5	0.5	2.5	0.5	1.25	2.5	0.375	0.125	0.5	1.0	0.125
		(S)	(S)	(S)	(S)	(S)	(S)	(S)	(S)	(S)	(S)	(S)	(S)
4	Fresh (1~3)	0.025	0.5	1.0	5.0	1.0	2.5	2.5	0.75	0.25	1.0	4.0	0.125
		(S)	(S)	(S)	(S)	(S)	(S)	(S)	(S)	(S)	(S)	(S)	(S)
	1	0.05	0.5	1.0	5.0	1.0	2.5	2.5	0.375	0.25	1.0	2.0	0.125
		(S)	(S)	(S)	(S)	(S)	(S)	(S)	(S)	(S)	(S)	(S)	(S)
	2	0.05	0.25	0.5	2.5	1.0	2.5	2.5	0.75	0.25	1.0	1.0	0.125
		(S)	(S)	(S)	(S)	(S)	(S)	(S)	(S)	(S)	(S)	(S)	(S)
	3	0.05	0.25	0.5	2.5	1.0	2.5	2.5	0.375	0.25	0.5	1.0	0.125
		(S)	(S)	(S)	(S)	(S)	(S)	(S)	(S)	(S)	(S)	(S)	(S)
6	Fresh (1~3)	0.05	0.25	0.5	2.5	1.0	1.25	2.5	0.375	0.25	1.0	2.0	0.125
		(S)	(S)	(S)	(S)	(S)	(S)	(S)	(S)	(S)	(S)	(S)	(S)
	1	0.05	0.25	0.25	2.5	0.5	1.25	1.25	0.375	0.125	0.5	1.0	0.125
		(S)	(S)	(S)	(S)	(S)	(S)	(S)	(S)	(S)	(S)	(S)	(S)
	2	0.05	0.25	0.25	2.5	0.5	2.5	1.25	0.375	0.125	0.5	1.0	0.125
		(S)	(S)	(S)	(S)	(S)	(S)	(S)	(S)	(S)	(S)	(S)	(S)
	3	0.025	0.25	0.25	2.5	0.5	1.25	2.5	0.375	0.125	0.5	2.0	0.125
		(S)	(S)	(S)	(S)	(S)	(S)	(S)	(S)	(S)	(S)	(S)	(S)

The 12 lyophilized anti-TB drugs in the DAC chip were validated by MIC determination using the clinical isolate KIT87190 strain. Activities of lyophilized drugs showed consistency at 4 °C for 6 months with quality control ranges of each drug. As a control, fresh drugs (Fresh) were prepared and the MIC values were determined. The tests were repeated three times.

**Table 2:** Comparison of validation results by the L-J method and the DAC system.

**Drug** (*μ*g/mL)	No. tested	Agreement (DAC/L-J)		Discrepancy (DAC/L-J)		Overall Agreement Rate	Sensitivity (%)	Specificity (%)	PPV (%)	NPV (%)
		R/R^a^	S/S^b^	R/S	S/R					
INH (0.1)	254	127	127	0	0	100% (254/254)	100	100	100	100
RIF (1.0)	254	124	129	0	1	99.6% (253/254)	99.2	100	100	99.2
STR (2.0)	254	65	171	15	3	92.9% (236/254)	95.6	91.9	81.3	98.3
EMB (5.0)	254	115	133	5	1	97.6% (248/254)	99.1	96.4	95.8	99.3
AMI (2.0)	254	79	168	4	3	97.2% (247/254)	96.3	97.7	95.2	98.2
CAP (2.5)	254	63	174	13	4	93.3% (237/254)	94.0	93.0	82.9	97.8
KAN (2.5)	254	89	155	9	1	96.1% (244/254)	98.9	94.5	90.8	99.4
LEV (1.5)	254	86	160	2	6	96.9% (246/254)	93.5	98.8	97.7	96.4
MOXI (0.5)	254	81	158	15	0	94.1% (239/254)	100	91.3	84.4	100
OFL (2.0)	254	96	156	0	2	99.2% (252/254)	98.0	100	100	98.7
PAS (4.0)	254	61	171	13	9	91.3% (232/254)	87.1	92.9	82.4	95
RFB (0.5)	254	91	156	4	3	97.2% (247/254)	96.8	97.5	95.8	98.1

We tested 254 MTB clinical isolates without measuring the inoculum sizes and all drugs were lyophilized in the DAC chip. Some of discrepant results were further analyzed via DNA sequencing (materials and methods).

^a^R: resistant, ^b^S: susceptible.

**Table 3:** Comparison of discrepant results after resolution by DNA sequence analysis.

Drug	No. Discrepancy (DAC/L-J)	No. tested by Sequencing	Agreement (DAC/Sequencing)	Agreement (L-J/Sequencing)
RIF	1	1	1/1 (100%)	0/1 (0%)
STR	18	11	7/11 (63.6%)	4/11 (36.4%)
EMB	6	4	2/4 (50.0%)	2/4 (50.0%)
AMI	7	7	4/7 (57.1%)	3/7 (42.9%)
CAP	17	13	9/13 (69.2%)	4/13 (30.8%)
KAN	10	3	1/3 (33.3%)	2/3 (66.7%)
LEV	8	7	5/7 (71.4%)	2/7 (28.6%)
MOXI	15	12	4/12 (33.3%)	8/12 (66.7%)
OFL	2	2	2/2 (100%)	0/2 (0%)
RFB	7	7	3/7 (42.9%)	4/7 (57.1%)

We analyzed 67 discrepant DST results by DNA sequencing for the target genes (Materials and Methods).

### Safety of the DAC system

**Figure 5:**
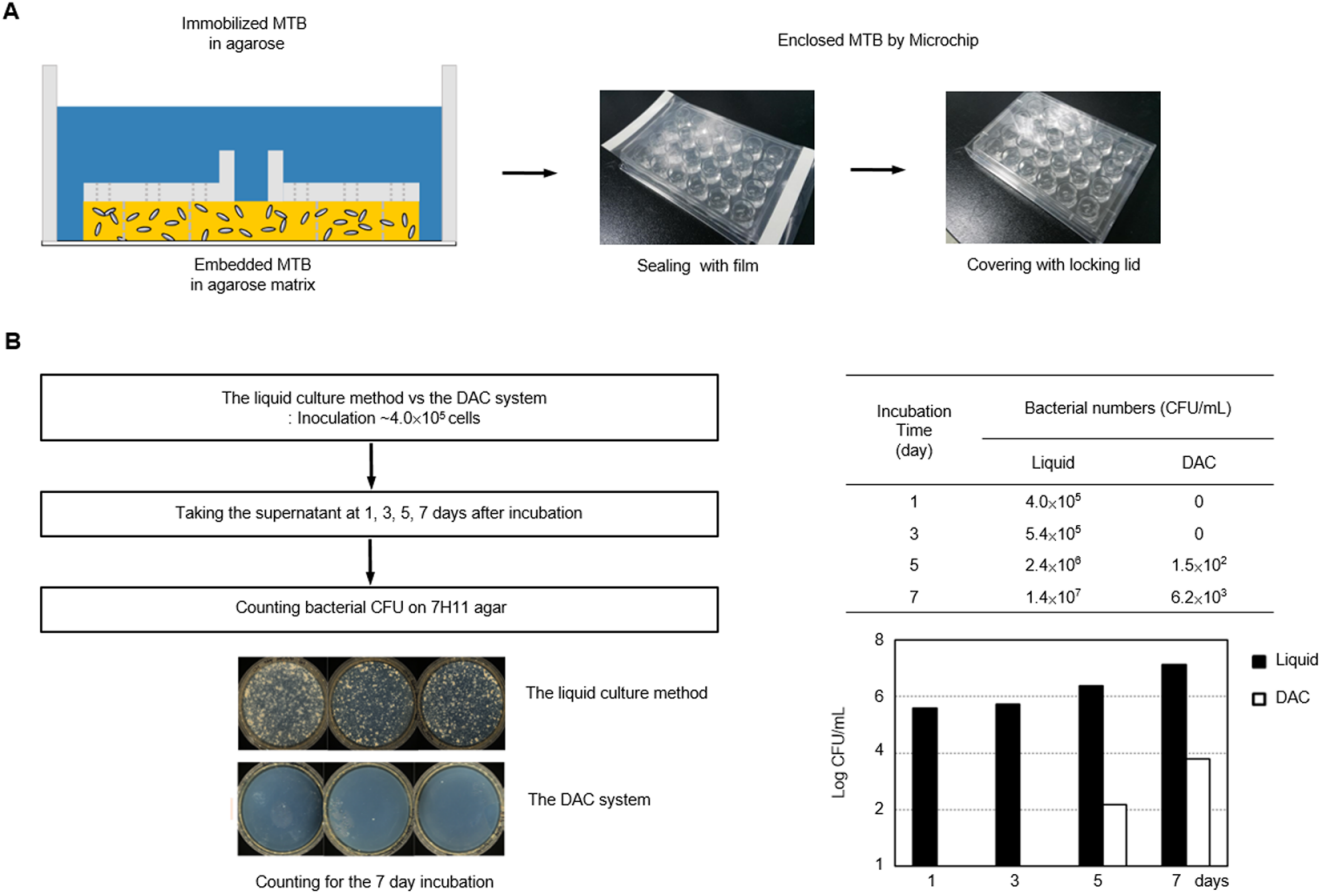
Safety of the DAC system. (**A**) The DAC system is safer for researchers as it blocks the leakage of MTB cells in three ways. (1) MTB cells in the DAC chip are immobilized in the solidified agarose matrix. (2) Each well in the chip is enclosed with sealing film and (3) the DAC chip is securely covered with a locking lid. **(B)** The comparison of MTB H37Rv cell numbers in the culture medium between the DAC chip and the liquid culture method after inoculation. MTB cells (~4.0 × 10^5^ CFU/mL) were inoculated both in the liquid culture system and in the DAC system. To perform this safety test, a 40-μL of the agarose-MTB mixture was loaded onto a DAC chip and 0.5 mL of the 7H9 medium was then added. After 1-, 3-, 5- and 7-day incubations, supernatants (50 μL) from both systems were collected using a pipette: The cells were plated on 7H11 agar plates, and 3 weeks later of culture, the bacterial cells were counted using the bacterial CFU method. In the DAC system, bacterial cells were not detected in culture broth until 1 and 3 incubation days, and there were ~1.5 × 10^2^ and ~6.2 × 10^3^ MTB cells on the 5th and 7th days, respectively. There were 1,000-fold fewer cells than in the liquid culture method.

In the MTB DST, safety is one of the most important issues. MTB cells can often infect lab researchers during DST procedures through MTB aerosol generation from serial dilution, or accidental leakage of MTB cells from test tubes or wells in a plate^30^. We had already demonstrated that the DAC system did not need serial dilution for preparation of the inoculum. The DAC system features extra safety devices, such as a sealing film and a locking lid. The MTB cells in the DAC system were embedded in the solidified agarose matrix, so that the suspended MTB cells could be minimized in the broth medium (Fig. 5A). Figure 5B shows the comparison of MTB cell counts between the DAC and the liquid culture systems in the broth medium. H37Rv was inoculated with 4 × 10^5^ CFU/mL into both the broth of the liquid culture system and the agarose matrix of the DAC system. At 1, 3, 5, and 7 days after inoculation, the supernatants from both devices were taken, and MTB cells were counted according to the CFU method. In the liquid culture system, MTB cells were observed in the broth after 1 day; the cell count increased from 4 × 10^5^ CFU/mL to 1.4 × 10^7^ CFU/mL. However, in the DAC system, MTB cells were detected in the broth only after 5 days, at 1.5 × 10^2^ CFU/mL; this count had slightly increased after 7 days, to 6.2 × 10^3^ CFU/mL. Taken together, the DAC system can efficiently prevent the accidental leakage of MTB cells during the DSTs to reduce TB infection risk.

## Discussion

There are some reasons that the MTB DSTs have not been performed easily and widely: (1) safety issues (2) irreproducible DST results and (3) a long turn-around time (4 to 6 weeks)^2,31^. The DAC system shows that it is possible to overcome these barriers. The MTB cells in the DAC system are embedded in the solidified agarose matrix, so that the suspended MTB cells can be minimized in the broth medium, to reduce the risk of contact from liquid medium leakage. The inoculum size is one of the main factors underlying these barriers, and inappropriate inoculum cell concentrations lead to DST errors^6,8,17^. The DAC system can produce faster and more reproducible DST results regardless of the inoculum size (in the range of 10^3^~10^8^ cell/mL) within 7 days. There was no inoculum effect in this system, and it was not necessary to measure McFarland turbidity values of the MTB cells to prepare the proper inoculum size.

In previous studies, the duration for which drug concentration remained above the MIC (% T > MICs) was measured in *in vivo* animal models, and there was no significant difference in %T > MIC required for drug efficacy, regardless of inoculum size or ESBL production status; on the other hand, the MIC values of *in vitro* tests were increased for high inoculum size^32^. This pattern suggested that the IE could be an artifact caused by the limitations of *in vitro* DST methods^26,27^. Based on this hypothesis, the DAC system could mimic *in vivo* DSTs more than the other conventional *in vitro* DST systems.

There are two possibilities why the inoculum effect did not occur in the DAC system: (1) the absolute amount of MTB cells is below the range in which the inoculum effect appears. In the DAC system, 10^3^~10^8^ cells/mL bacterial cell suspension is diluted with agarose at a 1:3 dilution ratio, and the agarose-cell solution contains 2.5 × 10^2^~2.5 × 10^7^ cells/mL of bacterial cells. The 40 μL mixture was loaded, and then 0.5 mL of liquid medium was added to the well. The final cell number for DST becomes 5.0 × 10^0^~5.0 × 10^5^ cells/mL. The inoculum size of 5.0 × 10^5^ cells/mL is hardly known to have an inoculum effect according to CLSI guidelines^18^. (2) The enclosed environmental conditions in the agarose matrix may contribute to the minimization of the inoculum effect. In the DAC system, the MTB cells are immobilized by agarose, and their metabolites or proteins that inhibit antibiotic activity could also be trapped in the agarose matrix, thus preventing easy binding and inactivation of antibiotics in the broth. In Fig. 3C, the result implies that beta-lactamase in the DAC system could be trapped in the agarose, and cannot bind and inactivate the antibiotic, even at high inoculum size.

The resolution results of the DAC system showed a better correlation with DNA sequencing when compared with the conventional method, but the cases (MOXI, KAN, and RFB) showed lower correlation by resolution (Table 3). For MOXI, four isolates showing the “R” agreement in the DAC system (4/12, 33.3%) were detected to have mutations in *gyrA*. The other eight isolates (“S” result in the L-J method and “R” in the DAC system) had a mutation in *gyrA* (S95T) as well, but this mutation is not associated with resistance^32^. To clarify MOXI sequencing results, it is necessary to analyze all regions associated with fluoroquinolone resistance, such as efflux pumps, *gyrB*, and the other regions of *gyrA*, despite showing low frequcency^33,34^. For RFB, three isolates showing “R” agreement in the DAC system (3/7, 42.9%) were detected as having SNPs causing S531L and H526Y in the *rpoB* region known as the resistant region. Among four discrepant isolates (“S” results in the DAC system), three were detected to have the same SNP (S531L), but one was observed to have an H516Y substitution in showing rare rifampicin or RFB susceptible isolates in the MGIT 960 DST^21^. For KAN, among 10 discrepant isolates, three isolates were tested by sequencing the *rrs*, *eis*, and *tlyA* regions. There was no mutation in three isolates, whereas mutations were detected in the *rrs* (nucleotide A1401G) region in two of these strains, no mutations were found in the *eis* and *tlyA* regions. Further studies may be necessary to understand the genetic basis of these phenotypes.

There are systems for rapid DSTs such as the MGIT 960 and MODS systems. The MGIT 960 system can generally produce DST results in 9 to 13 days and is commercialized, but this system has a severe inoculum effect (Fig. 2B) and detects cell growth by an indirect method, e.g., measuring fluorescence rates depending on the amount of oxygen consumption. In addition, the DST results are easily spoiled by bacterial contamination. In the microscopic observation drug susceptibility assay (MODS) system, MTB growth is measured by cord formation (a direct measuring method), and DST results can be produced in 5 to 14 days. However, it has been reported that some MTB cells cannot form cords^35^, and therefore, DST results are not reproducible. The MODS also poses a safety risk in the DST procedure. Therefore, the MODS system is not easily fully automated and has not yet been commercialized in the clinical area. The DAC system is fully automated and enables us to produce rapid and accurate MIC results regardless of the inoculum size and provides a safer DST process in one week, demonstrating that this system can be a better substitute for conventional DST systems. Although the DAC system has great potential to be used in the clinic, there remains some work to do. PZA is one of the first-line drugs but was not included in the DAC system because the DST culture condition for PZA is different from the other drugs. The DAC system needs a protocol for the PZA test so that it can be included. This system was validated with more than 250 clinical samples, but additional validation processes are needed with more clinical strains from various clinical sites.

## Electronic supplementary material

Supplementary Information
