# Supplementary material for: A rapid culture system uninfluenced by an inoculum effect increases reliability and convenience for drug susceptibility testing of Mycobacterium tuberculosis
Source: Sci Rep. 2018 Jun 5;8:8651. doi: 10.1038/s41598-018-26419-z (PMC5988837; doi:10.1038/s41598-018-26419-z)
Supplement: Supplementary file 1 — Supplementary Information [file 41598_2018_26419_MOESM1_ESM.doc]

**A rapid culture system uninfluenced by an inoculum effect increases reliability and convenience for drug susceptibility testing of *Mycobacterium tuberculosis***

Yong-Gyun Jung^1,2,3^†, Hyejin Kim^4^†, Sangyeop Lee^5^, Suyeoun Kim^5^, EunJi Jo^5^, Eun-Geun Kim^5^, Jungil Choi^6^, Hyun Jung Kim^5^ , Jungheon Yoo^5^, Hye-Jeong Lee^4^, Haeun Kim^4^, Hyunju Jung^4^, Sungweon Ryoo^9^*, Sunghoon Kwon^5,6,7,8^*

^1^ Interdisciplinary Program of Biomodulation, Myongji University, Yongin, Gyeonggi-do 17058, Republic of Korea.

^2^ Myongji Bioefficiency Research Centre, Myongji University, Yongin, Gyeonggi-do 17058, Republic of Korea.

^3^ Center for Nutraceutical and Pharmaceutical Materials, Myongji University, Yongin, Gyeonggi-do 17058, Republic of Korea.

^4^ Korean Institute of Tuberculosis, Osong, Cheongju, Chung Buk 28158, Republic of Korea.

^5^ QuantaMatrix Inc., Seoul National University Hospital CMI, Jongno-gu, Seoul 03082, Republic of Korea.

^6^ Department of Electrical Engineering and Computer Science, Seoul National University, Seoul 08826, Republic of Korea.

^7^ Institutes of Entrepreneurial BioConvergence, Seoul National University, Seoul 08826, Republic of Korea.

^8^ Seoul National University Hospital Biomedical Research Institute, Seoul National University Hospital, Seoul 03080, Republic of Korea.

^9^ Clinical Research Centre, Masan National Tuberculosis Hospital, Changwon, Korea.

*To whom correspondence should be addressed: E-mail: [skwon@snu.ac.kr](mailto:skwon@snu.ac.kr) (S.K.), [viweon@gmail.com](mailto:viweon@gmail.com) (S.R.)

†These authors contributed equally to this article.

**Supplementary Table S1**

| Anti-tuberculosis drug | Number of resistant strains |
| --- | --- |
| First-line drugs |  |
| INH | 141 |
| RIF | 138 |
| STR | 77 |
| EMB | 116 |
| Second-line drugs |  |
| AMI | 42 |
| CAP | 22 |
| KAN | 41 |
| LEV | 44 |
| MOXI | 41 |
| OFL | 46 |
| PAS | 26 |
| RFB | 39 |
| Total | 141 |
| Multidrug resistant MTB | 59 |
| Extensively resistant MTB | 82 |
|  | |

**Supplementary Table S1.** The CCs of five of the first-line and seven of the second-line anti-TB drugs in the DAC system were determined with 230 clinical isolates whose DST results were already well characterized by both the absolute L-J method and DNA sequence analysis.

**Supplementary Table S2.**

| Week | Drug (μg/mL) | | INH | RIF | STR | EMB | AMI | CAP | KAN | LEV | MOXI | OFL | PAS | RFB |
| --- | --- | --- | --- | --- | --- | --- | --- | --- | --- | --- | --- | --- | --- | --- |
|  | Batch | # Replicate |  |  |  |  |  |  |  |  |  |  |  |  |
| 1 | 1 | # 1 | 0.025 | 0.125 | 0.5 | 2.5 | 0.5 | 1.25 | 1.25 | 0.375 | 0.125 | 0.5 | 1.0 | 0.125 |
|  |  | # 2 | 0.025 | 0.125 | 0.5 | 2.5 | 0.5 | 1.25 | 1.25 | 0.375 | 0.125 | 0.5 | 1.0 | 0.125 |
|  |  | # 3 | 0.05 | 0.25 | 0.5 | 2.5 | 0.5 | 1.25 | 1.25 | 0.375 | 0.125 | 0.5 | 1.0 | 0.125 |
|  | 2 | # 1 | 0.025 | 0.25 | 0.5 | 2.5 | 0.5 | 0.625 | 1.25 | 0.375 | 0.125 | 0.5 | 1.0 | 0.125 |
|  |  | # 2 | 0.025 | 0.25 | 0.5 | 2.5 | 0.5 | 0.625 | 1.25 | 0.375 | 0.125 | 0.5 | 1.0 | 0.125 |
|  |  | # 3 | 0.05 | 0.25 | 0.25 | 2.5 | 0.25 | 1.25 | 1.25 | 0.375 | 0.125 | 0.5 | 1.0 | 0.125 |
|  | 3 | # 1 | 0.05 | 0.25 | 0.5 | 2.5 | 0.5 | 1.25 | 1.25 | 0.375 | 0.125 | 0.5 | 1.0 | 0.125 |
|  |  | # 2 | 0.025 | 0.5 | 1.0 | 2.5 | 1.0 | 2.5 | 2.5 | 0.75 | 0.25 | 1.0 | 2.0 | 0.125 |
|  |  | # 3 | 0.05 | 0.25 | 0.5 | 2.5 | 0.5 | 1.25 | 1.25 | 0.375 | 0.125 | 0.5 | 1.0 | 0.25 |
|  | 4 | # 1 | 0.05 | 0.25 | 0.5 | 2.5 | 0.5 | 1.25 | 1.25 | 0.375 | 0.125 | 0.5 | 1.0 | 0.25 |
|  |  | # 2 | 0.025 | 0.25 | 0.5 | 2.5 | 1.0 | 1.25 | 1.25 | 0.375 | 0.125 | 0.5 | 1.0 | 0.125 |
|  |  | # 3 | 0.05 | 0.25 | 0.5 | 2.5 | 0.5 | 1.25 | 1.25 | 0.375 | 0.125 | 0.5 | 1.0 | 0.125 |
|  | 5 | # 1 | 0.05 | 0.25 | 0.5 | 2.5 | 0.5 | 1.25 | 1.25 | 0.375 | 0.125 | 0.5 | 1.0 | 0.125 |
|  |  | # 2 | 0.025 | 0.25 | 0.5 | 2.5 | 0.5 | 0.625 | 1.25 | 0.375 | 0.125 | 1.0 | 1.0 | 0.125 |
|  |  | # 3 | 0.05 | 0.25 | 0.5 | 2.5 | 0.5 | 1.25 | 1.25 | 0.375 | 0.125 | 0.5 | 1.0 | 0.25 |
| 2 | 1 | # 1 | 0.05 | 0.25 | 0.5 | 2.5 | 0.5 | 1.25 | 1.25 | 0.375 | 0.125 | 0.5 | 1.0 | 0.125 |
|  |  | # 2 | 0.05 | 0.25 | 0.5 | 2.5 | 0.5 | 1.25 | 1.25 | 0.375 | 0.125 | 0.5 | 1.0 | 0.125 |
|  |  | # 3 | 0.05 | 0.5 | 0.5 | 2.5 | 1.0 | 2.5 | 2.5 | 0.375 | 0.25 | 0.5 | 4.0 | 0.125 |
|  | 2 | # 1 | 0.05 | 0.25 | 0.5 | 2.5 | 0.5 | 1.25 | 1.25 | 0.375 | 0.125 | 0.5 | 1.0 | 0.125 |
|  |  | # 2 | 0.025 | 0.25 | 0.5 | 2.5 | 1.0 | 2.5 | 2.5 | 0.375 | 0.25 | 1.0 | 1.0 | 0.125 |
|  |  | # 3 | 0.05 | 0.25 | 0.5 | 2.5 | 0.5 | 1.25 | 1.25 | 0.375 | 0.125 | 0.5 | 1.0 | 0.125 |
|  | 3 | # 1 | 0.05 | 0.25 | 0.5 | 2.5 | 0.5 | 1.25 | 1.25 | 0.375 | 0.125 | 0.5 | 1.0 | 0.125 |
|  |  | # 2 | 0.05 | 0.25 | 0.5 | 2.5 | 0.5 | 1.25 | 1.25 | 0.375 | 0.125 | 0.5 | 1.0 | 0.125 |
|  |  | # 3 | 0.05 | 0.25 | 0.25 | 2.5 | 1.0 | 1.25 | 0.625 | 0.375 | 0.0625 | 0.5 | 1.0 | 0.125 |
|  | 4 | # 1 | 0.05 | 0.25 | 0.5 | 2.5 | 0.5 | 1.25 | 1.25 | 0.375 | 0.125 | 0.5 | 1.0 | 0.125 |
|  |  | # 2 | 0.05 | 0.25 | 0.5 | 2.5 | 0.5 | 0.625 | 1.25 | 0.375 | 0.125 | 0.5 | 1.0 | 0.125 |
|  |  | # 3 | 0.05 | 0.25 | 0.5 | 2.5 | 0.5 | 1.25 | 1.25 | 0.375 | 0.125 | 0.5 | 1.0 | 0.125 |
|  | 5 | # 1 | 0.05 | 0.25 | 0.5 | 2.5 | 0.5 | 0.625 | 1.25 | 0.375 | 0.125 | 0.5 | 1.0 | 0.125 |
|  |  | # 2 | 0.05 | 0.25 | 0.5 | 2.5 | 0.5 | 0.625 | 1.25 | 0.375 | 0.125 | 0.5 | 1.0 | 0.125 |
|  |  | # 3 | 0.05 | 0.25 | 0.5 | 1.25 | 0.5 | 0.625 | 1.25 | 0.375 | 0.125 | 0.5 | 1.0 | 0.125 |

**Supplementary Table S2. Determination of the quality control range of MIC for each drug.** The quality control range of the 12 lyophilized anti-TB drugs in the DAC chip were validated using the clinical isolate KIT87190 strain. The tests were repeated in three different batches, five times per week, for 2 weeks, resulting in 30 replicates.

**Supplementary Table S3.**

| Week | Drug (μg/mL) | INH | RIF | STR | EMB | AMI | CAP | KAN | LEV | MOXI | OFL | PAS | RFB |
| --- | --- | --- | --- | --- | --- | --- | --- | --- | --- | --- | --- | --- | --- |
|  | Batch |  |  |  |  |  |  |  |  |  |  |  |  |
| 1 | # 1 | 0.05 | 0.125 | 1.0 | 2.5 | 1.0 | 1.25 | 2.5 | 0.375 | 0.125 | 0.5 | 1.0 | 0.125 |
|  | # 2 | 0.05 | 0.125 | 1.0 | 2.5 | 1.0 | 1.25 | 2.5 | 0.375 | 0.125 | 0.5 | 1.0 | 0.125 |
|  | # 3 | 0.05 | 0.125 | 1.0 | 2.5 | 1.0 | 1.25 | 2.5 | 0.375 | 0.125 | 0.5 | 1.0 | 0.125 |
|  | # 4 | 0.05 | 0.125 | 0.5 | 1.25 | 1.0 | 2.5 | 2.5 | 0.375 | 0.125 | 0.5 | 1.0 | 0.125 |
|  | # 5 | 0.05 | 0.125 | 0.5 | 2.5 | 1.0 | 1.25 | 2.5 | 0.375 | 0.125 | 0.5 | 1.0 | 0.125 |
| 2 | # 1 | 0.05 | 0.125 | 0.5 | 2.5 | 1.0 | 1.25 | 2.5 | 0.375 | 0.125 | 0.5 | 1.0 | 0.125 |
|  | # 2 | 0.025 | 0.125 | 0.5 | 1.25 | 0.5 | 1.25 | 1.25 | 0.375 | 0.125 | 0.5 | 1.0 | 0.125 |
|  | # 3 | 0.025 | 0.125 | 0.5 | 1.25 | 0.5 | 1.25 | 1.25 | 0.375 | 0.125 | 0.5 | 1.0 | 0.125 |
|  | # 4 | 0.05 | 0.125 | 1.0 | 2.5 | 1.0 | 1.25 | 2.5 | 0.375 | 0.125 | 0.5 | 1.0 | 0.125 |
|  | # 5 | 0.05 | 0.125 | 1.0 | 2.5 | 1.0 | 1.25 | 2.5 | 0.375 | 0.125 | 0.5 | 1.0 | 0.125 |
| 3 | # 1 | 0.05 | 0.25 | 1.0 | 2.5 | 0.5 | 2.5 | 2.5 | 0.375 | 0.25 | 1.0 | 1.0 | 0.125 |
|  | # 2 | 0.05 | 0.125 | 1.0 | 2.5 | 1.0 | 1.25 | 2.5 | 0.375 | 0.125 | 0.5 | 1.0 | 0.125 |
|  | # 3 | 0.05 | 0.125 | 1.0 | 2.5 | 1.0 | 1.25 | 2.5 | 0.375 | 0.125 | 0.5 | 1.0 | 0.125 |
|  | # 4 | 0.05 | 0.125 | 1.0 | 2.5 | 1.0 | 1.25 | 2.5 | 0.375 | 0.125 | 0.5 | 1.0 | 0.125 |
|  | # 5 | 0.05 | 0.125 | 1.0 | 2.5 | 1.0 | 1.25 | 2.5 | 0.375 | 0.125 | 0.5 | 1.0 | 0.125 |

**Supplementary Table S3. Reproducibility test of the DAC system.** The lyophilized drug in the DAC chip was tested five times by three different operators during three different weeks, using the H37Ra reference strain, resulting in 15 replicates. The activities of each drug were obtained reproducibly.
